# Identification of PAFAH1B3 as Candidate Prognosis Marker and Potential Therapeutic Target for Hepatocellular Carcinoma

**DOI:** 10.3389/fonc.2021.700700

**Published:** 2021-08-19

**Authors:** Weikang Xu, Xinyu Lu, Jing Liu, Qianhui Chen, Xuan Huang, Kuiyuan Huang, Hongyan Liu, Wei Zhu, Xiaoyong Zhang

**Affiliations:** ^1^State Key Laboratory of Organ Failure Research, Guangdong Provincial Key Laboratory of Viral Hepatitis Research, Department of Infectious Diseases, Nanfang Hospital, Southern Medical University, Guangzhou, China; ^2^Department of Hematology, The Seventh Affiliated Hospital of Sun Yat-sen University, Shenzhen, China

**Keywords:** PAFAH1B3, hepatocellular carcinoma, biomarker, prognosis, cancer databases

## Abstract

**Background:**

Hepatocellular carcinoma (HCC) is the fourth leading cause of cancer-related deaths worldwide. PAFAH1B3 plays an important role on occurrence and development in a variety tumor. However, the function of PAFAH1B3 in HCC remains unclear.

**Methods:**

The TIMER, ONCOMINE, Human Protein Atlas (HPA), GEPIA, The Cancer Genome Atlas (TCGA), HCCDB, UALCAN and LinkedOmics database were used to analyze the prognostic value, co-expression genes and regulator networks of PAFAH1B3 in HCC. siRNA transfections and inhibitor of PAFAH1B3 P11 were used to verify the anti-tumor effect on HCC cell lines. Gene expression was detected by qRT-PCR. The functions of PAFAH1B3 downregulation in HCC cell lines were investigated using cell cycle analysis, apoptosis detection, CCK8 assay and transwell assay. Western blot was used to evaluate the role of PAFAH1B3 on metabolic pathways in HCC cells.

**Results:**

Based on the data from databases, the expression of PAFAH1B3 was remarkably increased in HCC patients. High expression of PAFAH1B3 was associated with poorer overall survival (OS) and disease-free survival (DFS). And PAFAH1B3 was notably linked to age, sex, grade, stage, race, and TP53 mutational status. Then, the functional network analysis showed PAFAH1B3 may be involved in HCC through cell cycle, cell metabolism, spliceosome, and RNA transport. Furthermore, the mRNA expression of PAFAH1B3 was also increased in HCC cell lines. Flow cytometry analysis showed that PAFAH1B3 manipulated apoptosis and cell cycle regulation. CCK8 assay showed that PAFAH1B3 silencing or pharmacologic inhibitor of PAFAH1B3 inhibited the proliferation of HepG2, Huh7 and MHCC-97H cells. Transwell assay results showed that PAFAH1B3 silencing also significantly impaired the invasion and migratory ability of HCC cells. In addition, PAFAH1B3 silencing significantly downregulated the expression of glycolysis and lipid synthesis signaling pathways.

**Conclusion:**

Our findings suggested that PAFAH1B3 plays a critical role in progression of HCC. PAFAH1B3 as a prognosis marker and potential target for HCC has prospective clinical significance.

## Introduction

Primary liver cancer is one of the fourth most common malignant tumors leading to death, hepatocellular carcinoma (HCC) is the most common form of primary liver cancer and is an important medical problem (1, 2). The development of HCC is closely related to the existence of chronic liver disease, the risk factors leading to HCC are known to mainly include hepatitis B virus (HBV), hepatitis C virus (HCV), alcohol, metabolic syndrome, diabetes, obesity, nonalcoholic fatty liver disease (NAFLD) and so on (2, 3). HCC treatment options vary and depend on tumor stages. For the early and intermediate stage, surgical resection image-guided ablation and chemoembolization treatments are mainly (2). However, most patients are diagnosed in advanced stages when HCC is discovered and are characterized by poor survival (4, 5). It’s because there is a lack of effective biomarkers to detect and treat HCC. Most recently, the FDA approved sorafenib, cabozantinib, lenvatinib and other drugs for the treatment of advanced HCC (6–8), but the overall survival of patients is still less than 1 year (9). Therefore, we urgently need to explore new oncogenes, especially those that can be used as targets for the treatment of HCC.

Platelet-activating factor acetyl hydrolase (PAF-AH), an acetylhydrolase, is known to induce the inactivation of the platelet-activating factor (PAF) through deacetylation (10). PAF-AH contains 3 subunits: PAFAH1B1, PAFAH1B2, and PAFAH1B3, with PAFAH1B2 and PAFAH1B3 exhibiting catalytic functions (11). Several studies have indicated the relationship between PAFAH1B3 and cancer progression. Blocking PAFAH1B3 and PAFAH1B2 have been shown to extensively affect lipid metabolism and upregulate the expression of lipids that inhibit breast cancer growth (12). Selective inhibition of PAFAH1B3 has been reported remarkably increased in gastric cancer cells, and high PAFAH1B3 expression was significantly correlated with proliferation, migration and immune infiltration (13). A previous study showed that PAFAH1B3 intervention might be useful for the treatment of HCC (14). However, the specific biological role of PAFAH1B3 in HCC and whether it is a therapeutic target remains unclear.

In our study, we used multiple cancer databases to analyze the PAFAH1B3 expression in various cancer, and further analyzed the prognostic value, co-expression genes, regulator networks of PAFAH1B3 in HCC. Importantly, further investigation *in vitro* revealed the correlation of PAFAH1B3 with proliferation, apoptosis, cell cycle, metastasis, and tumor-infiltrating immune cells. Finally, to further explore the potential relationship between PAFAH1B3 and tumor metabolic activity, we tested the expression of key targets involved in lipid and glucose metabolism. Our finding provides new insights into potential prognosis and therapeutic strategies for HCC.

## Materials and Methods

### ONCOMINE Dataset

The ONCOMINE online cancer database (https://www.oncomine.org/) was used to compare the transcription levels of PAFAH1B3 in multiple cancers (15). Several forms of HCC studies, including the Roessler Liver, Roessler Liver 2, Chen Liver, and Wurmbach Liver Study, were used in this analysis (16–18). This database allows the comparison of the relative transcript levels of PAFAH1B3 in cancer specimens and normal controls (15). The cutoff p-value was set as 0.01, and the fold change was set as 1.5.

### GEPIA Dataset

GEPIA (http://gepia.cancer-pku.cn/) is a new web server for analyzing the gene expression profiling of 9736 cancer and 8587 normal samples produced by TCGA and Genotype-Tissue Expression (GTEx) projects (19). This database allows the analysis of differential gene expression, pathological stages, patient survival curves, and evaluation of similar genes and correlation in tumor and normal samples (19).

### Human Protein Atlas Analysis

HPA (https://www.Proteinatlas.org/) was used to analyze the protein expression of PAFAH1B3 in normal and HCC tissues (20).

### HCCDB Dataset

HCCDB is an HCC online database for exploring the gene expression of 3917 clinical samples based on TCGA and GTEx. HCCDB provides functions such as differential gene expression analysis, survival analysis, and coexpression analysis (21).

### UALCAN Dataset

The UALCAN (http://ualcan.path.uab.edu) interactive web portal was used to analyze the gene expression profiles of 31 cancer types based on TCGA and clinical samples (22). UALCAN allows the analysis of relative gene expression, clinicopathological features, and differential gene expression in individual cancer types (22).

### LinkedOmics Dataset

LinkedOmics (23) is a multiomics database that integrates global proteomics data into TCGA tumor samples. It contains multiomics and clinical data for 32 cancer types and 11 158 clinical samples based on the TCGA project. LinkedOmics provides 3 analysis modules, namely the LinkFinder, LinkCompare, and LinkInterpreter modules, to conduct a comprehensive analysis of the data. The cutoff FDR was set as 0.05, and the simulation was set to 1000. LinkedOmics is publicly available at (http://www.linkedomics.org/login.php).

### TIMER Analysis

TIMER dataset (http://timer.comp-genomics.org/) was used to investigate the correlation between the expression of PAFAH1B3 and infiltration levels of immune cells (24).

### Cell Culture and Transfection

Cell lines were acquired from the Cell Bank of the Type Culture Collection (Chinese Academy of Sciences, Shanghai, China). HepG2, MHCC-97H, Huh7, and LO2 were cultured in DMEM (Gibco, USA) supplemented with 10**%** fetal bovine serum (Gibco, USA) and antibiotics at 37**°**C and 5**%** CO2. HCC cell lines were transfected with siRNAs targeting PAFAH1B3 or control siRNA (Genechem, China) using Lipofectamine RNAIMAX (Invitrogen, USA). The efficiency of the knockdown was confirmed using qRT-PCR analysis. The siRNA used were as follows: PAFAH1B3 siRNA1: 5**’**- CGACAGGUGAACGAGCUGGUATT-3**’**; PAFAH1B3 siRNA2: 5**’**-GGAGAAGAACCGACAGGUGAATT-3**’**.

### Growth Inhibition Assays

For the *in vitro* studies, P11 (Cayman, USA), a selective inhibitor of PAFAH1B3, was dissolved in DMSO at a concentration of 50 mM, kept at -20°C as a stock solution. For the analysis of cell lines, HepG2, Huh7 and MHCC-97H cells were grown in drug-free medium for 3 days prior to experiences. The HCC cells were treated with various concentrations of P11 at -37°C for 48h. cells were treated DMSO instead of P11 served as control group. The experience was performed at least 3 times.

### Quantitative Real Time Polymerase Chain Reaction (qRT-PCR)

Total RNA was isolated from HepG2, MHCC-97H, Huh7, and LO2 cell lines using the One Step TB Green^®^ PrimeScript™ PLUS RT-PCR Kit (Takara, Beijing, China) according to the manufacturer’s protocol. All samples were run on a Roche LightCycler 480 machine. The cycles of target genes were normalized to those of the β-actin gene to obtain the ΔCT value. The expression of each target gene was presented as fold change, which was calculated through relative quantification using the 2−ΔΔCt method. The primers used were as follows: PAFAH1B3: Forward: 5’-ACATCCGGCCCAAGATTGTG-3’; Reverse: 3’-GGGCTGTCGCTCATTCACC-5. PKM2: Forward: 5-’ATGTCGAAGCCCCATAGTGAA-3’; Reverse: 3’-TGGGTGGTGAATCAATGTCCA-5’. GLUT1: Forward: 5’-GCCAGAAGGAGTCAGGTTCAA-3’; Reverse: 3’-TCCTCGGAAAGGAGTTAGATCC-5’. PFK: Forward: 5’-GTACCTGGCGCTGGTATCTG-3’; Reverse: 3’-CCTCTCACACATGAAGTTCTCC-5’. HK2: Forward: 5’-GAGCCACCACTCACCCTACT-3’; Reverse: 3’-CCAGGCATTCGGCAATGTG-5’. FASN: Forward: 5’-AAGGACCTGTCTAGGTTTGATGC-3’; Reverse: 3’-TGGCTTCATAGGTGACTTCCA-5’. SREBP-1: Forward: 5’-TGCTCTGGCTTTGCCTTGCTG-3’; Reverse: 3’-AGGGCGTGAAGACTGAGGTGGA-5’. β-actin: Forward: 5’-CATGTACGTTGCTATCCAGGC-3’; Reverse: 3’-CTCCTTAATGTCACGCACGAT-5’.

### Cell Counting Kit-8 (CCK8) Assay

The proliferation of HCC cells was assessed using the CCK-8 assay (Takara, China). Briefly, 2000 cells/well were incubated in triplicate in a 96-well plate, and 10 μL CCK-8 was added to each well after 48 h. After a 4h incubation at 37**°**C, the optical density (OD) value was measured at 450 nm absorbance.

### Western Blot

For western blot analysis, the total cellular protein of HCC cells was obtained by RIPA lysis buffer. The protein concentration was determined using the bicinchoninic acid (BCA) assay (Fdbio Science, China). Equal amounts of protein sample were loaded to sodium dodecyl sulfate-polyacrylamide gel electrophoresis (SDS–PAGE) and then transferred to the nitrocellulose filter membrane (GE Healthcare, USA). Next, membranes were incubated with the blocking buffer Tris-buffered saline/Tween 20 (TBST) in 5% bovine calf serum at room temperature for 1 hour and then incubated with primary antibody (CSTC64G5, CST4970L, ab150299, ab3259) overnight at 4°C and horseradish peroxidase (HRP)-conjugated secondary antibody. Signals of the protein were detected using an ImageQuant LAS 4000 auto-exposure system (GE Healthcare).

### Transwell Assay

The transwell assay were using the 8.0μM transwell chamber (Corning, USA). DMEM medium without FBS was placed into the upper chambers to rehydrate the Matrigel (Corning, USA) for 2 hours at 37°C. Then remove the supernatants, one hundred thousand HCC cells containing DMEM medium without FBS were plated in the upper chamber. After incubation for 48 hours, the cells in the upper chambers were fixed with 4% paraformaldehyde for 15 minutes and stained with crystal violet for 20 minutes. The numbers of invading and migrating cells were counted in three randomly selected fields.

### Flow Cytometry Analysis

We analyzed the apoptosis and cell cycle of HCC cell lines using Flow Cytometry. After indicated treatments, for the cell apoptosis analysis, HCC cells were collected separately, and incubated with Annexin-V (BioLegend, United States) in the dark for 15 min, the apoptotic cells were detected on a FACSCantoTM II Flow Cytometer (BD, United States). For the cell cycle analysis, HCC cells were collected separately, and resuspend in 1 mL of ice-cold ethanol 70% overnight at 4°C, then incubated with RNaseA (Novoprotein, China) at 37°C for 30 min, then incubated with propidium iodide (PI) in the dark at 4°C for 30 min, then scanned in FACSCantoTM II Flow Cytometer.

### Statistical Analysis

We performed differential gene expression and DNA copy number analyses between tumor and normal samples using the t-test. The p-value and fold change were used to assess the statistical significance of the difference, with p < 0.01 and fold change >1.5 being considered significant. We also used t-test to conduct analysis of multiple clinicopathological characteristics of samples of patients with HCC from UALCAN, with p < 0.05 indicating significance. Kaplan-Meier curves were used to analyze the prognosis of patients with HCC with different median expression levels of PAFAH1B3. A log-rank test p < 0.05 indicated that the analysis was statistically significant. All experience data were analyzed using GraphPad 8.0.

## Result

### Elevated Expression of PAFAH1B3 in a Variety of Malignant Tumors

**Figure 1:**
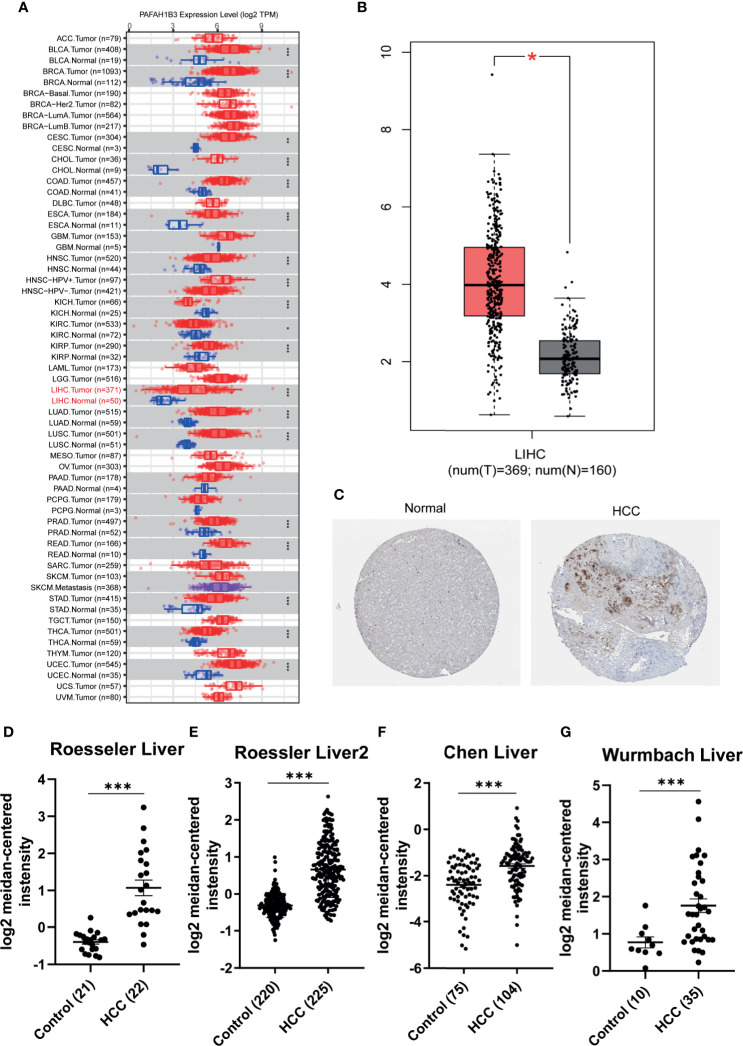
Expression of PAFAH1B3 in hepatocellular carcinoma (HCC). **(A)** The transcription level of PAFAH1B3 in TIMER databases. **(B)** The transcription level of PAFAH1B3 in ONCOMINE databases. **(C)** The protein expression of PAFAH1B3 in LIHC (HPA). **(D)** Boxplot showing the levels of PAFAH1B3 mRNA in the Roessler Liver. **(E)** Boxplot showing the levels of PAFAH1B3 mRNA in the Roessler Liver 2 datasets. **(F)** Boxplot showing the levels of PAFAH1B3 mRNA in the Chen Liver datasets. **(G)**. Boxplot showing the levels of PAFAH1B3 mRNA in the Wurmbach Liver datasets. *p < 0.05; ***p < 0.001.

We used the ONCOMINE, TIMER and HCCDB cancer database to analyze the mRNA expression of PAFAH1B3 in multiple cancer types. Our results show that PAFAH1B3 was highly expressed in a wide variety of malignant tumors compared with normal samples, including BLCA (P=3.20E-09), BRCA (P=3.20E-09). CHOL (P=2.04E-64), COAD (P=2.26E-09), ESCA (P=2.11E-07), HNSC (P=4.15E-13), KICH (P=5.04E-09), KIRC (P=0.041), KIRP (P=9.92E-06), LIHC (P= 4.51E-20), LUAD (P=7.89E-35), LUSC (P=2.01E-31), PRAD (P=2.52E-13), READ (P=1.57E-07), STAD (P=1.69E-15), THCA (P=1.98E-22), UCEC (P=2.52E-13) (**Figure 1A**). The protein and mRNA expression of PAFAH1B3 was also highly expressed in HCC tissues (**Figures 1B, C** and **Table 1**). Several forms of HCC studies, including the Roessler Liver, Roessler Liver 2, Chen Liver, and Wurmbach Liver Study, also showed similar results (**Figures 1D–G** and **Supplement Table 1**). We observed that multiple clinicopathological analyses of PAFAH1B3 in the UALCAN database consistently showed the elevated transcription levels of PAFAH1B3 in HCC patients than healthy people in subgroup analyses based on age, gender, tumor grade, cancer stages, ethnicity and TP53 mutation (**Table 2**).

**Table 1:** The expression of PAFAH1B3 in tumor tissues and the adjacent normal tissues, according to t-test in HCCDB.

Dataset	P-value	Type	Nums	Mean	STD	IQR
HCCDB1	2.28E-14	HCC	100	8.697	1.106	1.585
		Adjacent	97	7.683	0.3789	0.4513
HCCDB3	1.37E-27	HCC	268	0.9672	0.7824	0.7668
		Adjacent	243	0.3788	0.1215	0.153
		Cirrhotic	40	0.5489	0.1984	0.3387
		Healthy	6	0.2823	0.056	0.0575
HCCDB4	5.03E-66	HCC	240	8.834	1.126	1.511
		Adjacent	193	7.092	0.461	0.5144
HCCDB6	3.23E-48	HCC	225	5.121	0.7661	1.168
		Adjacent	220	4.143	0.3332	0.4088
HCCDB7	0.0000237	HCC	80	12.09	0.6094	0.7463
		Adjacent	82	11.68	0.5761	0.824
HCCDB11	0.05348	HCC	88	11.72	1.617	1.973
		Adjacent	48	11.14	1.683	1.822
HCCDB12	0.00000334	HCC	81	8.451	1.15	1.257
		Adjacent	80	7.73	0.6739	0.7978
HCCDB13	4.25E-26	HCC	228	5.658	0.9374	1.326
		Adjacent	168	4.884	0.2996	0.3545
HCCDB15	1.76E-22	HCC	351	8.412	1.483	1.835
		Adjacent	49	6.967	0.6521	1
HCCDB16	0.001937	HCC	60	8.14	0.458	0.5118
		Adjacent	60	7.904	0.3497	0.4085
HCCDB17	0.002157	HCC	115	9.009	1.536	1.65
		Adjacent	52	8.496	0.5865	0.68
HCCDB18	1.23E-37	HCC	212	3.017	1.111	1.353
		Adjacent	177	1.737	0.569	0.62

**Table 2:** Expression of PAFAH1B3 in LIHC (Liver hepatocellular carcinoma) subgroups classified by age, sex, tumour grade, individual cancer stages, race, and TP53 mutational status (UALCAN).

Clinicopathological characteristics	N	P-value
Age (Yrs)		
21-40	27	1.75E-05
41-60	140	5.68E-07
61-80	181	<1E-12
81-100	10	5.14E-03
Gender		
Male	245	8.89E-10
Female	117	1.62E-12
Grade		
1	54	3.63E-05
2	173	1.62E-12
3	118	8.39E-07
4	12	1.50E-03
Stage		
1	168	1.62E-12
2	84	4.50E-14
3	82	2.04E-04
4	6	3.31E-02
Race		
Caucasian	177	<1E-12
African-american	17	8.76E-04
Asian	157	5.11E-08
TP53 mutation		
Yes	105	3.32E-07
NO	255	1.62E-12

### High Expression of PAFAH1B3 Is Associated With Poor Prognosis in HCC

**Figure 2:**
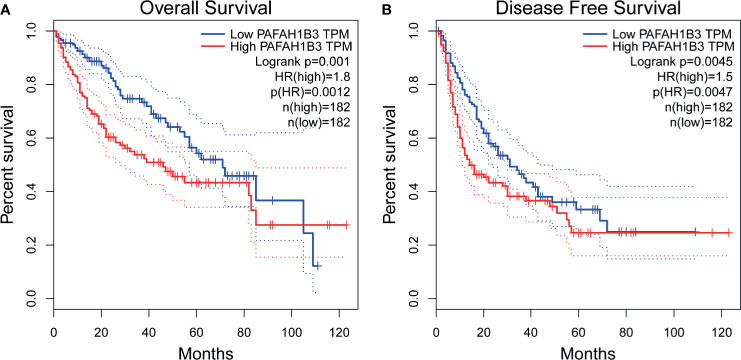
Prognostic Value of PAFAH1B3 in Patients with hepatocellular carcinoma (HCC) (GEPIA). **(A)** Overall survival (OS) of PAFAH1B3 in HCC. **(B)** Disease-free survival (DFS) of PAFAH1B3 in HCC.

We evaluated the prognostic value of PAFAH1B3 in a variety of malignant tumors using the GEPIA database. Patients were divided into two groups based on the median expression level of PAFAH1B3 in each cohort. We found that the increased expression of PAFAH1B3 was correlated with poor overall survival (OS) (P=0.0012) and disease-free survival (DFS) (P=0.0047) in HCC (**Figures 2A, B**). Besides, high expression of PAFAH1B3 was also associated with overall survival in adrenocortical carcinoma (P=0.00035), acute myeloid leukemia (P=0.032), lung adenocarcinoma (P=0.008), mesothelioma (P=0.0022), sarcoma and skin cutaneous Melanoma (P=0.0038) (**Supplement Figures 1A–F**).

### Coexpression Genes of PAFAH1B3 in Hepatocellular Carcinoma

**Figure 3:**
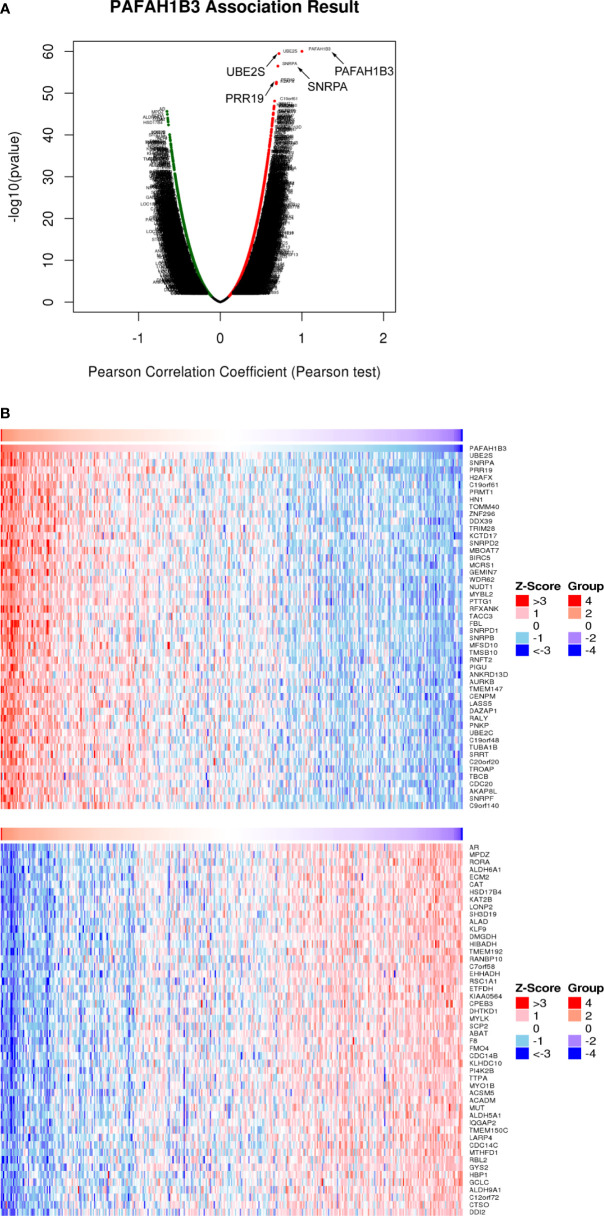
Coexpression of PAFAH1B3 in hepatocellular carcinoma (HCC) (LinkedOmics). **(A)** Total genes highly correlated with PAFAH1B3. **(B)** Heatmaps showing the 50 significant genes positively and negatively correlated with PAFAH1B3 in LIHC. Red and blue indicate positively and negatively correlated genes, respectively.

We examined the genes coexpressed with PAFAH1B3 to explore common genetic risk factors in HCC. As shown in **Figure 5A**, 19 922 genes were shown to be significantly associated with PAFAH1B3. More specifically, several genes, such as PRR19, UBE2S, and SNRPA, showed a strong positive association with PAFAH1B3 (**Figure 3A** and **Supplement Table 2**). We found that the identified coexpressed genes were involved in cell cycle regulation (25), promotion of tumor growth, metastasis, and recurrence (26). **Figure 3B** shows the 50 significant genes positively and negatively correlated with PAFAH1B3 respectively. Then, co-expressed genes enrichment analysis was using Gene Ontology (GO) and Kyoto Encyclopedia of Genes and Genomes (KEGG) databases. The Go analysis results showed PAFAH1B3 co-expressed genes participate primarily in mitotic cell cycle phase transition, RNA catabolic processes, mRNA processing pathways (**Supplement Figure 2A**). The KEGG analysis results showed that spliceosome, ribosome, cell cycle and RNA transport were significantly enriched (**Supplement Figure 2B**). These results suggest that a widespread impact of PAFAH1B3 on the proliferation.

### Regulators of PAFAH1B3 Networks in Hepatocellular Carcinoma

To further explore the targets of PAFAH1B3 in HCC, the generated kinase, miRNA, and transcription factor target networks of positively correlated gene sets were analyzed by GSEA. Our results showed that the ataxia telangiectasia mutated and Rad3-related (ATR), cyclin-dependent kinase 1 (CDK1), and polo-like kinase 1 (PLK1) kinases were the most significant target networks related to kinase-target regulation (**Table 3**). Moreover, these screened kinase genes were shown to be significantly related to HCC (27–29). We further noticed that the miRNA-target network was associated with (ATGTTAA) MIR-302C, (TAATAAT) MIR-126, (TGAATGT) MIR-181A, MIR-181B, MIR-181C, and MIR-181D (**Table 3**). The enrichment of transcription factors was shown to be related mainly to E2F, ELK1, and NRF1 (**Table 3**). We also observed that the identified transcription factors were also significantly related to HCC (30–32).

**Table 3:** The Kinase, miRNA and transcription factor-target networks of PAFAH1B3 in HCC (LinkedOmics).

Enriched category	Geneset	LeadingEdgeNum	FDR
Kinase Target	Kinase_ATR	23	0
	Kinase_CDK1	81	0
	Kinase_PLK1	34	0
	Kinase_PRKCI	12	0
miRNA Target	ATGTTAA,MIR-302C	69	0.0019025
	TAATAAT,MIR-126	70	0.0019025
	TGAATGT,MIR-181A,MIR-181B,MIR-181C,MIR-181D	145	0.014269
	GCACTTT,MIR-17-5P,MIR-20A,MIR-106A,MIR-106B,MIR-20B,MIR-519D	163	0.016684
	ATATGCA,MIR-448	71	0.017938
	TTTGCAC,MIR-19A,MIR-19B	164	0.018679
	ATGTACA,MIR-493	105	0.021404
	GTGCAAT,MIR-25,MIR-32,MIR-92,MIR-363,MIR-367	94	0.023918
	ATACTGT,MIR-144	73	0.024071
Transcription Factor	V$E2F_Q6_01	77	0.0027256
	SCGGAAGY_V$ELK1_02	307	0.012898
	RCGCANGCGY_V$NRF1_Q6	237	0.014989
	TGCGCANK_UNKNOWN	136	0.018564

### PAFAH1B3 mRNA Was Upregulated in Hepatocellular Carcinoma Cell Lines, PAFAH1B3 Silencing or Inhibitor P11 Impairing HepG2, Huh7 and MHCC-97H Proliferation and Metastasis

**Figure 4:**
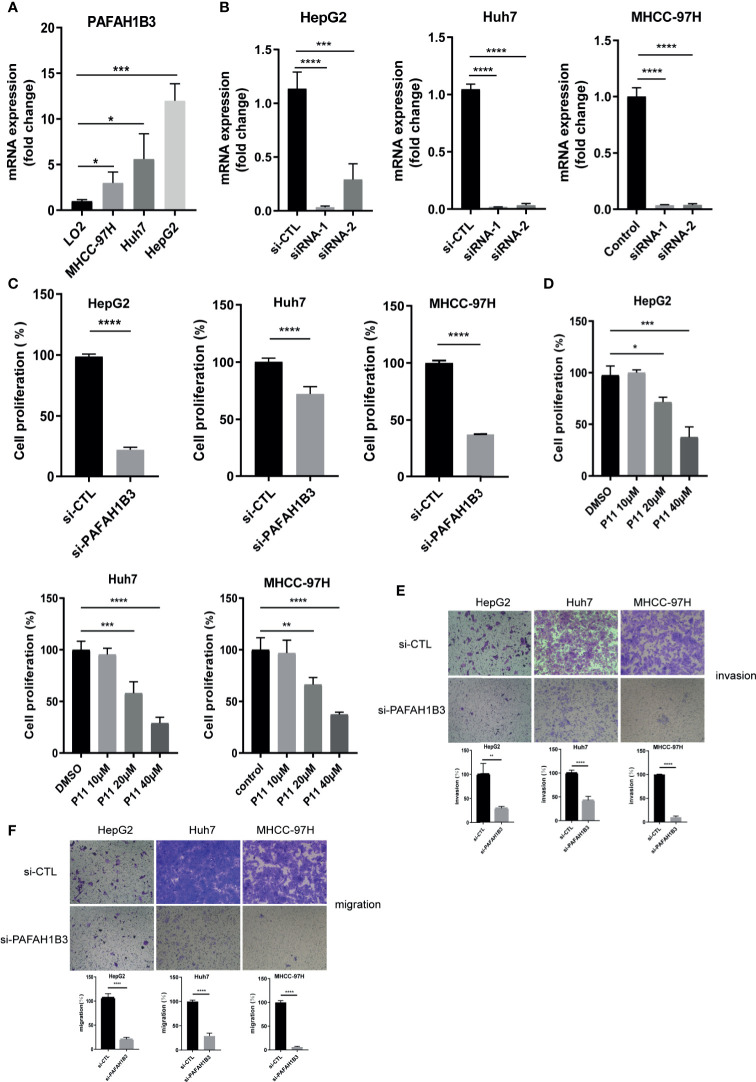
High expression of PAFAH1B3 related to the proliferation and metastasis of HCC cells. **(A)** Levels of PAFAH1B3 mRNA in LO2, MHCC-97H, Huh7, and HepG2 cell lines. **(B)** Quantitation of the levels of PAFAH1B3 mRNA in HepG2, Huh7 and MHCC-97H cells by qRT-PCR after transfection for 48 h with different siRNAs targeting PAFAH1B3 (si-1 and si-2) or a control siRNA. **(C)** Proliferation of HepG2 and Huh7 cells after silencing PAFAH1B3 for 48 h **(D)** Proliferation of HepG2 and Huh7 cells after pharmacologic inhibitor of PAFAH1B3 for 48 h **(E)** Invasion assay. **(F)** Migratory assay. *p < 0.05; **p < 0.01; ***p < 0.001; ****p < 0.0001.

Firstly, we verified the mRNA expression of PAFAH1B3 in HCC cell lines. We found that the levels of mRNA expression of PAFAH1B3 were increased in HCC cell lines, especially in HepG2, compared with the LO2 liver cell line (p < 0.001) (**Figure 4A**). We verified that PAFAH1B3 was successfully silenced by siRNA in HepG2, Huh7 cells and MHCC-97H (p<0.0001) (**Figure 4B**). Next, we use the CCK8 assay to explore the effects of PAFAH1B3 silencing and pharmacological inhibition on proliferation of HCC cells. Our results showed that siRNA-induced silencing impaired the proliferation of HepG2, Huh7 and MHCC-97H cells (p<0.0001) (**Figure 4C**). P11 could also impaired the proliferation of HepG2, Huh7 and MHCC-97H cells in a concentration dependent manner (**Figure 4D**). Besides, transwell assay results showed that PAFAH1B3 silencing also significantly impaired the invasion and migratory ability of HCC cells (**Figures 4E, F**). These results suggested that high expression of PAFAH1B3 was associated with the proliferation and metastasis of HCC cell lines.

### PAFAH1B3 Silencing and P11 Induce Cell Cycle Arrest and Apoptosis

**Figure 5:**
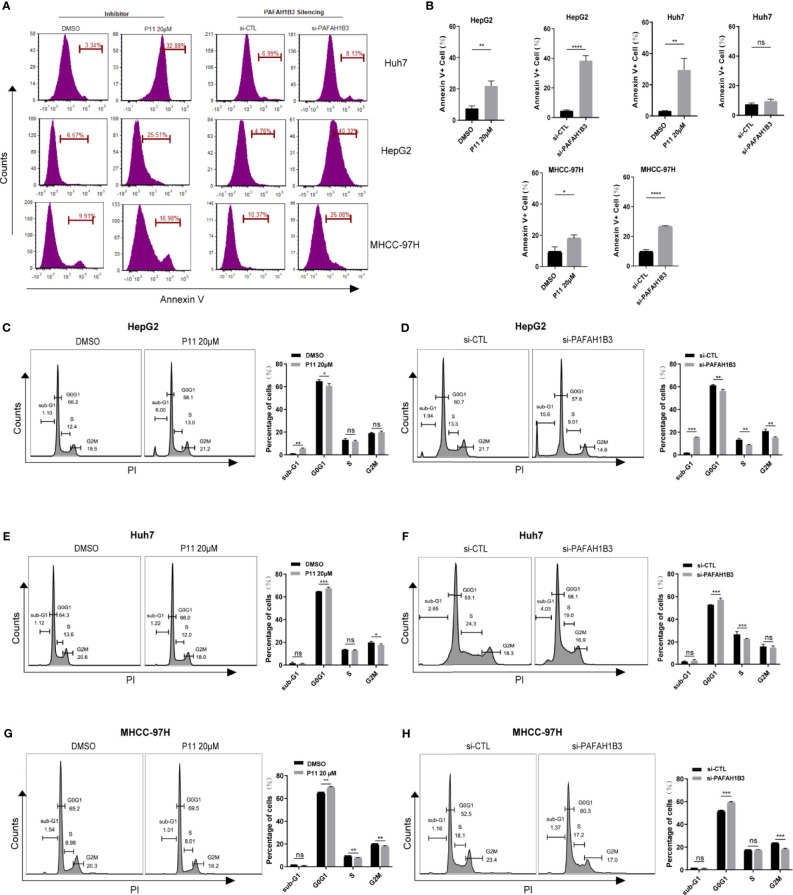
The effects of PAFAH1B3 on HCC cell apoptosis and cell cycle. **(A, B)** The flow cytometry analysis revealed the effects of PAFAH1B3 silencing or inhibitor P11 on cell apoptosis. **(C–H)** Cell cycle analysis in HepG2, Huh7 and MHCC-97H cells. *p < 0.05; **p < 0.01; ***p < 0.001; ****p < 0.0001. NS, no significance.

Then we further explore the effects of PAFAH1B3 on distribution of the cell cycle and apoptosis in HCC cells by flow cytometric analysis. PAFAH1B3 was silenced by siRNA or pharmacological inhibited by P11 with 20μM dose for 48 hours. As shown in **Figures 5A, B**, P11 was significantly induced apoptosis in HepG2, huh7 and MHCC-97H cells, PAFAH1B3 siRNA was only induced apoptosis in HepG2 and MHCC-97H, but not in Huh7. P11, a selective inhibitor of PAFAH1B3, also could target PAFAH1B2. Therefore, we used ONCOMINE online cancer database to analyze the mRNA expression of PAFAH1B1 and PAFAH1B2 in HCC and found that the mRNA expression of PAFAH1B1 and PAFAH1B2 was no significant changes in HCC (**Supplement Figure 3**). So, we did not continue to study the role of PAFAH1B2 in HCC, but our results showed that both PAFAH1B2 and PAFAH1B3 inhibition could have better effector than PAFAH1B3 siRNA in Huh7 cells. Next, we found that PAFAH1B3 silencing or P11 increased the percentage of HepG2 in sub-G1 phase (**Figures 5C, D**). PAFAH1B3 silencing or P11 induced G0/G1 cell cycle arrest in Huh7 and MHCC-97H (**Figures 5E–H**). These results implied that targeting PAFAH1B3 exerts potent antitumor effects in HCC cell lines by inducing cell cycle arrest and apoptosis.

### PAFAH1B3 Silencing Downregulated the Expression of Glycolysis and Lipid Synthesis Signaling Pathways

**Figure 6:**
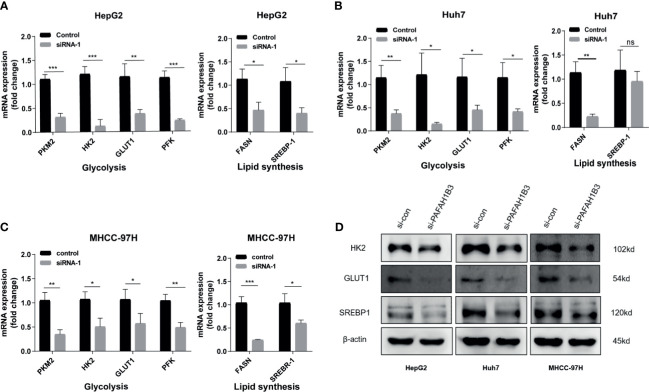
PAFAH1B3 downregulates glycolysis and lipid synthesis signaling pathways. **(A–C)** Analysis of glycolysis-and lipid synthesis-related genes (PKM2, HK2, GLUT1, PFK, FASN and SREBP-1) in HepG2 Huh7 and MHCC-97H cells after silencing PAFAH1B3 for 48 h. **(D)** Analysis of glycolysis-related key enzymes and lipid synthesis-related transcription factor (HK2, GLUT1 and SREBP-1) in HCC cells by Western blot after silencing PAFAH1B3 for 48 h. *p < 0.05; **p < 0.01; ***p < 0.001. NS, no significance.

We next investigated whether the metabolic genes of HCC were regulated by PAFAH1B3 silencing in HepG2, Huh7 and MHCC-97H cells. In the present study, qRT-PCR showed glycolysis- and lipid synthesis-related genes, including PKM2, HK2, GLUT1, PFK, FASN and SREBP-1, were significantly downregulated by the silencing of PAFAH1B3 in HepG2, Huh7, and MHCC-97H cells (**Figures 6A–C**). Finally, we verified the impact of PAFAH1B3 on lipid synthesis and glycolysis signaling pathways by western blot. The results show that PAFAH1B3 Silencing in HepG2, Huh7 and MHCC-97H led to the obviously downregulated levels of Glut1. And the levels of HK2 and SREBP1 were slightly downregulated (**Figure 6D**). Taken together, the PAFAH1B3 participated in maintaining metabolic homeostasis in HCC cells.

## Discussion

HCC is one of the most common cancers in the world, there is currently no effective treatment for HCC. Therefore, new therapeutic targets to treat HCC are urgently needed. In recent years, PAFAH1B3, as a pan-cancer target, is found to be significantly upregulated in a variety of malignant tumors. Recent studies showed PAFAH1B3 as a key metabolic driver was correlated with poor prognosis, and PAFAH1B3 knockdown or pharmacological inhibition impaired cancer cell survival through enhancing tumor-suppressing signaling lipids (33, 34). Similarly, PAFAH1b3 was also highly expressed in gastric cancer cells and osteosarcoma cells, and PAFAH1B3 knockdown was impaired cancer cell proliferation (13, 35). However, the biological role of PAFAH1b3 in HCC remains unclear.

Firstly, we demonstrated that PAFAH1B3 was highly expressed in a wide variety of malignant tumors compared with normal samples. And, high expression of PAFAH1B3 was also associated with overall survival. Besides, high expression of PAFAH1B3 predicted the poor prognosis of patients with HCC and showed that it was related to tumor stage and survival rate. Our results showed that PAFAH1B3 can be used as a pan-cancer marker for tumor prognosis.

To further study the role of PAFAH1B3 in HCC, we used the LinkedOmics function module to analyze PAFAH1B3 co-expressed genes to further explore the biological significance of PAFAH1B3 in HCC. We accordingly found that related functional networks were involved in cell cycle, cell metabolism, spliceosome, and RNA transport. Co-expression genes have been shown to be involved in protein degradation and signal transduction, promoting proliferation of multiple tumors, such as melanoma (36) and gastric cancer (37). We assessed the regulators of PAFAH1B3 in HCC by analyzing the enrichment of kinases, miRNAs, and transcription factors (TF) of PAFAH1B3 positively related genes and found that PAFAH1B3 was primarily related to the CDK1 and PLK1 kinases in HCC (**Supplement Table 1**). Overexpression of CDK1 and PLK1 has been reported to cause malignant transformation of hepatocytes, defining a subgroup of high-risk patients (29, 38). The E2F family has been implicated as the main transcription factor in the abnormal regulation of PAFAH1B3, with the E2F signal being shown to interact with the transcription program to promote the development of HCC (39).

Then, we verified the mRNA expression of PAFAH1B3 in MHCC-97H, Huh7, HepG2, and LO2 cells. We found that the mRNA expression of PAFAH1B3 in HCC cell lines was higher than that in LO2. We anticipated that these results would be useful for improving current treatment regimens and outcomes of HCC. To further explore whether PAFAH1B3 can be used as a target for the treatment of HCC, we tested the proliferation of HepG2, Huh7 and MHCC-97H cells after silencing or pharmacologic inhibitor of PAFAH1B3. On the one hand, our results suggested that the proliferation of HepG2, Huh7 and MHCC-97H was significantly suppressed after silencing or pharmacologic inhibitor of PAFAH1B3. On the other hand, PAFAH1B3 silencing significantly impaired HCC cells migration and invasion potential, suggesting that PAFAH1B3 plays an important role in maintaining the aggressive properties of HCC cells. In line with our findings, it has been reported that PAFAH1B3 knockdown suppressed cells proliferation and impaired cells metastasis in breast cancer and gastric cancer (13, 33). A recent study showed that palmitoylation of PAFAH1b3 was required to promote cell migration and PAFAH1b3 knockdown abrogated the ability of VEGF (and insulin) to stimulate angiogenesis (40). Next, we found that targeting PAFAH1B3 could significantly induce cell cycle arrest in HCC cells and induce apoptosis in HCC cells. PAF-AH contains 3 subunits: PAFAH1B1, PAFAH1B2, and PAFAH1B3. However, we found that the mRNA expression of PAFAH1B1 and PAFAH1B2 was no significant changes in HCC. In line with our findings, PAFAH1B2 was no significantly upregulated in human breast tumors, but targeting PAFAH1B2 could also significantly impaired proliferation, survival, migration, and invasiveness in breast cancer cells (34). So, the functions of PAFAH1B2 and PAFAH1B3 need further exploration.

In addition, PAFAH1B3 silencing significantly downregulated Glycolysis and lipid metabolism signaling pathway. PAFAH1B3, a newly discovered metabolic enzyme, was correlated with infiltration of immune cells in HCC (**Supplement Figure 4**). Metabolomics analysis revealed that PAFAH1B3 promotes tumor invasion by regulating the precancer signaling lipid metabolism network; thus, targeting PAFAH1B3 was shown to upregulate the pathogenicity of tumor suppressor lipids to attenuate cancer (12, 34). Several studies have shown that targeting metabolic enzymes could effectively inhibit tumor growth and promote infiltration of T-cells, thus rendering tumors more responsive to immune checkpoint therapy (41–43). Our early study showed that adoptive transfer of the AFP TCR-T could eradicate HepG2 tumors in NSG mice (44). Whether targeting PAFAH1B3 can affect the anti-tumor immune response remains to be verified.

## Conclusion

In summary, our study demonstrated that PAFAH1B3 has important prognosis value as a biomarker for HCC. By targeting PAFAH1B3, the proliferation and metastasis of HepG2, Huh7 and MHCC-97H cells was inhibited. Mechanistically, we found PAFAH1B3 was involved in regulation of cell cycle, apoptosis, and metabolism in HCC cell lines. Altogether, PAFAH1B3 may be used as a prognostic biomarker and potential therapy target for HCC.

## Data Availability Statement

The datasets presented in this study can be found in online repositories. The names of the repository/repositories and accession number(s) can be found in the article/**Supplementary Material**.

## Author Contributions

XZ, WZ, and HL conceived and supervised the study. WX, XL, and JL designed experiments. WX, XL, QC, and XH performed experiments. XL, JL, QC, and KH analyzed data. WX wrote the manuscript. XZ, WZ, and HL made manuscript revisions. All authors reviewed the results. All authors contributed to the article and approved the submitted version.

## Funding

This study was supported by National Natural Science Foundation of China (grant number 81802449; 81871664).

## Conflict of Interest

The authors declare that the research was conducted in the absence of any commercial or financial relationships that could be construed as a potential conflict of interest.

## Publisher’s Note

All claims expressed in this article are solely those of the authors and do not necessarily represent those of their affiliated organizations, or those of the publisher, the editors and the reviewers. Any product that may be evaluated in this article, or claim that may be made by its manufacturer, is not guaranteed or endorsed by the publisher.

## Supplementary Material

Supplementary Figure 1 Prognostic Value of PAFAH1B3 in various cancer types. Overall survival of PAFAH1B3 was analyzed using GEPIA database in **(A)** adrenocortical carcinoma. **(B)** acute myeloid leukemia, **(C)** lung adenocarcinoma, **(D)** mesothelioma, **(E)** sarcoma, **(F)** skin cutaneous Melanoma.

Supplementary Figure 2 Gene ontology annotations and KEGG pathways enriched with PAFAH1B3 Co-expressed genes in hepatocellular carcinoma (LinkedOmics). GSEA was used to analyze the significantly enriched GO_BP, and KEGG pathways of PAFAH1B3 Co-expressed genes in LIHC. **(A)** Gene ontology biological processes, analyzed by GSEA. **(B)** KEGG pathway, analyzed by GSEA. The LeadingEdgeNum and the false discovery rate (FDR) are shown in blue and orange columns, respectively.

Supplementary Figure 3 Expression of PAFAH1B1 and PAFAH1B2 in HCC.

Supplementary Figure 4 Correlation between PAFAH1B3 expression and immune cell infiltration levels in HCC tissues analyzed *via* TIMER.

Supplementary Table 1 The statistics of PAFAH1B3 expression in LIHC based on ONCOMINE.

Supplementary Table 2 The top 50 co-expressed genes of PAFAH1B3 (LinkedOmics).
