# Supplementary figures and images for: Identification of PAFAH1B3 as Candidate Prognosis Marker and Potential Therapeutic Target for Hepatocellular Carcinoma
Source: Front Oncol. 2021 Aug 19;11:700700. doi: 10.3389/fonc.2021.700700 (PMC8418329; doi:10.3389/fonc.2021.700700)

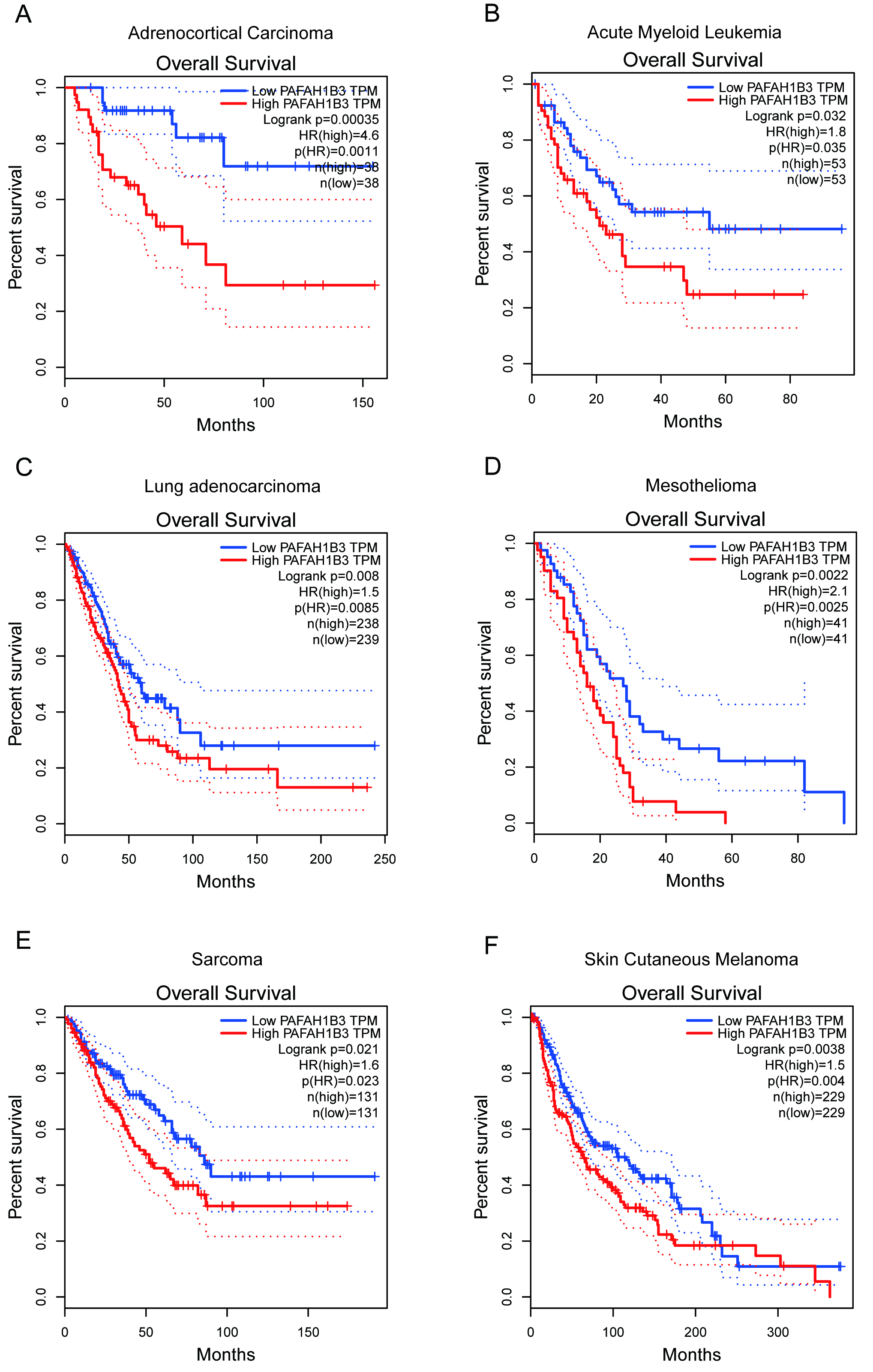

Supplement: Supplementary Figure 1 — Prognostic Value of PAFAH1B3 in various cancer types. Overall survival of PAFAH1B3 was analyzed using GEPIA database in (A) adrenocortical carcinoma. (B) acute myeloid leukemia, (C) lung adenocarcinoma, (D) mesothelioma, (E) sarcoma, (F) skin cutaneous Melanoma. [file Image_1.tif]

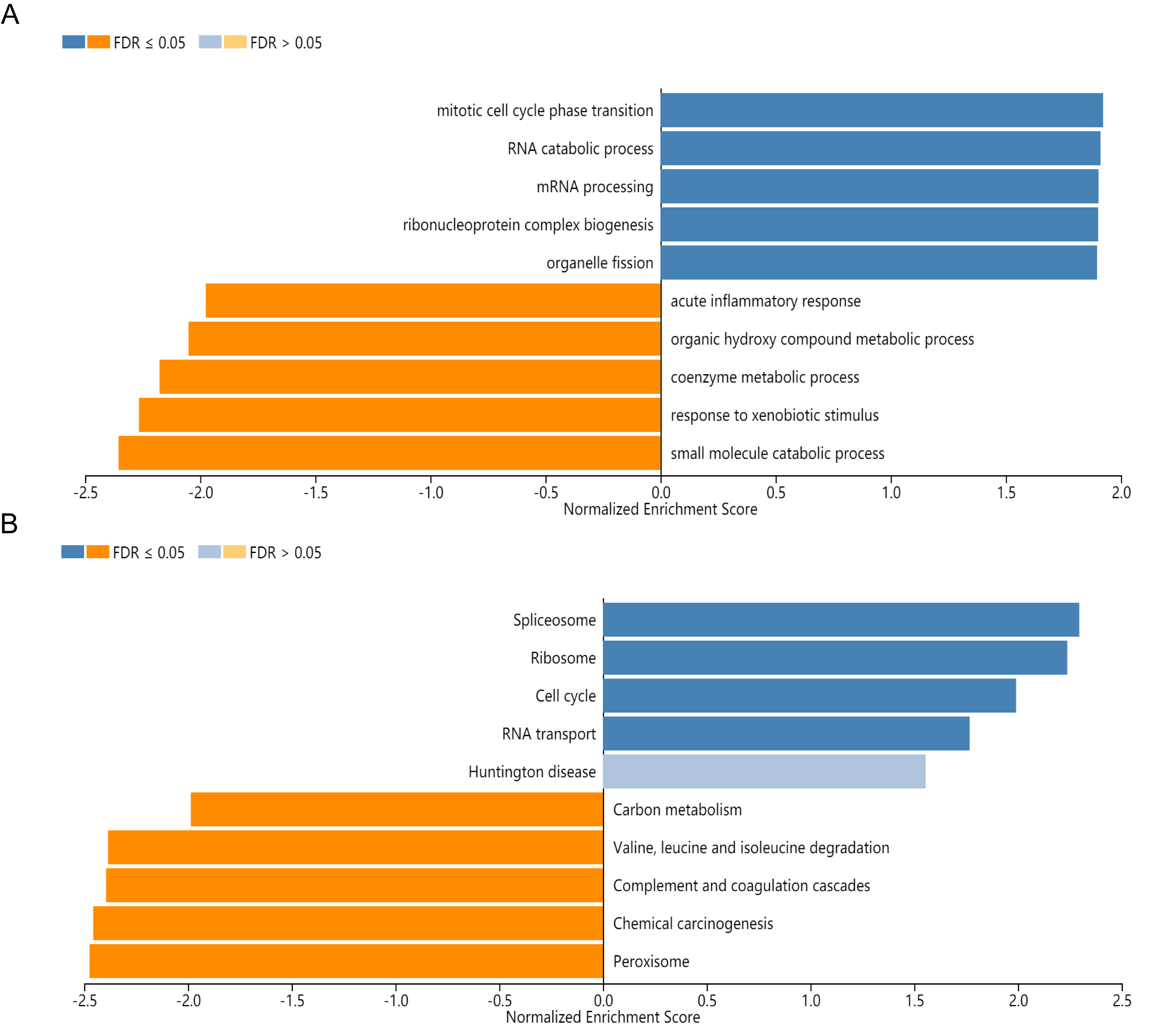

Supplement: Supplementary Figure 2 — Gene ontology annotations and KEGG pathways enriched with PAFAH1B3 Co-expressed genes in hepatocellular carcinoma (LinkedOmics). GSEA was used to analyze the significantly enriched GO_BP, and KEGG pathways of PAFAH1B3 Co-expressed genes in LIHC. (A) Gene ontology biological processes, analyzed by GSEA. (B) KEGG pathway, analyzed by GSEA. The LeadingEdgeNum and the false discovery rate (FDR) are shown in blue and orange columns, respectively. [file Image_2.tif]

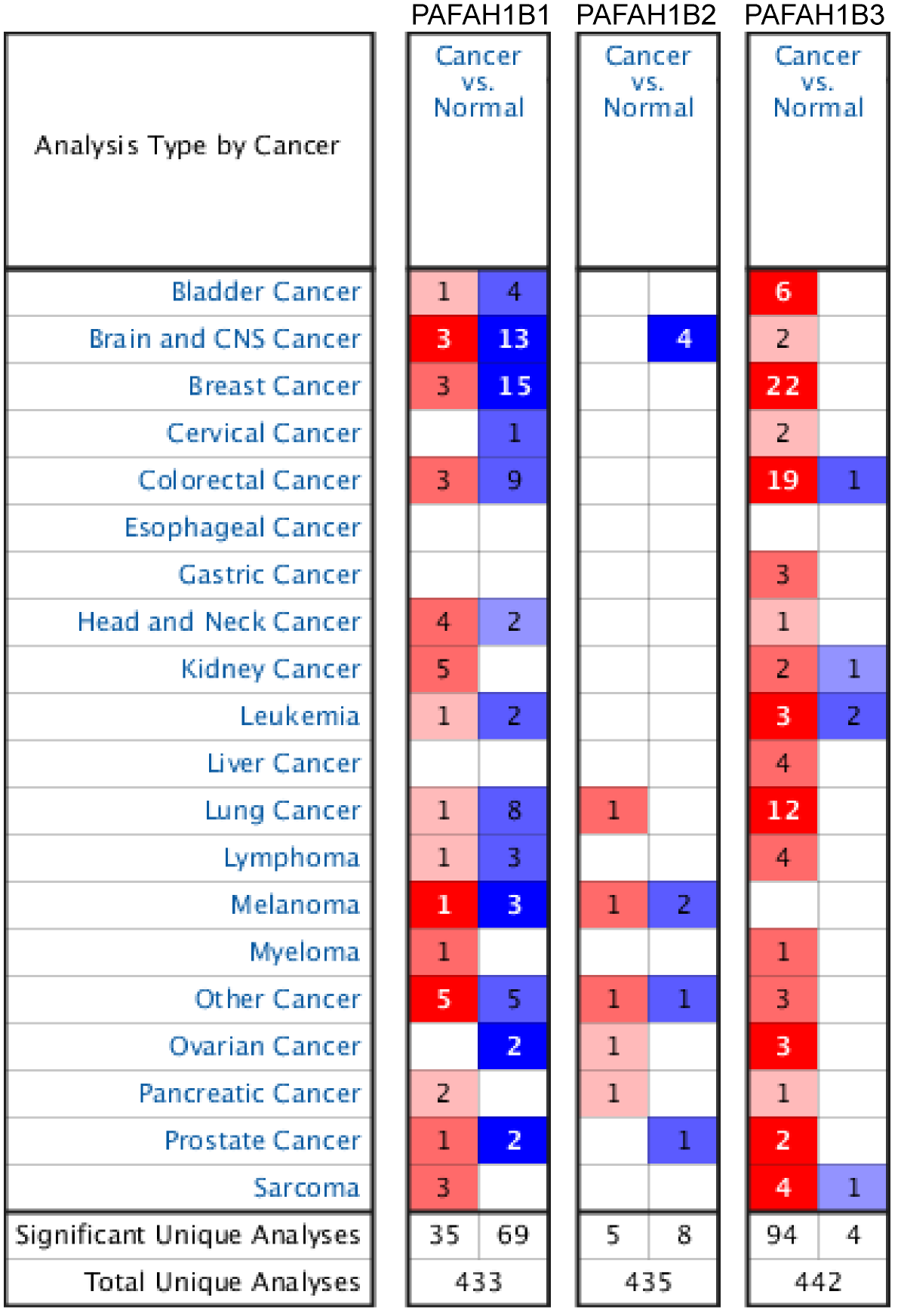

Supplement: Supplementary Figure 3 — Expression of PAFAH1B1 and PAFAH1B2 in HCC. [file Image_3.tif]

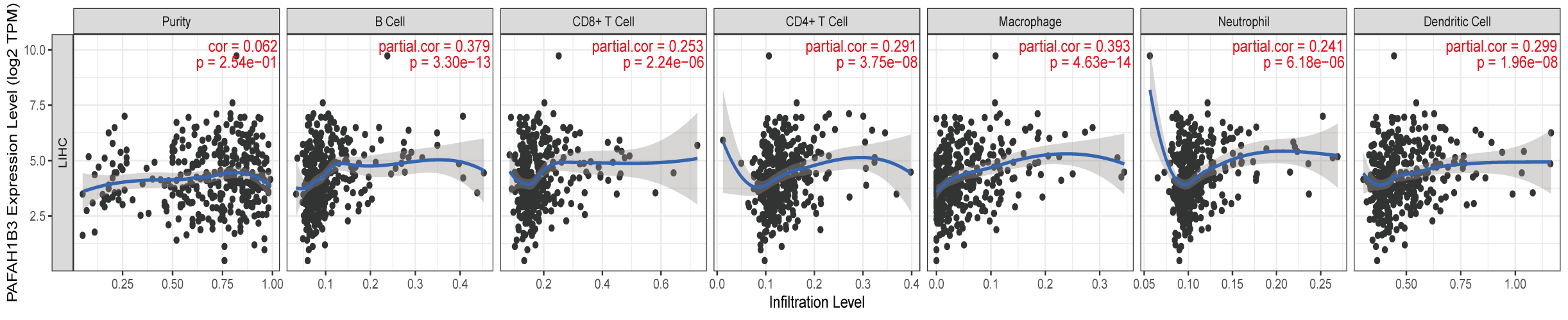

Supplement: Supplementary Figure 4 — Correlation between PAFAH1B3 expression and immune cell infiltration levels in HCC tissues analyzed via TIMER. [file Image_4.tif]
