# Supplementary material for: Identification of PAFAH1B3 as Candidate Prognosis Marker and Potential Therapeutic Target for Hepatocellular Carcinoma
Source: Front Oncol. 2021 Aug 19;11:700700. doi: 10.3389/fonc.2021.700700 (PMC8418329; doi:10.3389/fonc.2021.700700)
Supplement: Supplementary Table 1 — The statistics of PAFAH1B3 expression in LIHC based on ONCOMINE. [file Table_1.docx]

**Supplementary Table 1.** The statistics of PAFAH1B3 expression in LIHC based on ONCOMINE.

| Dataset | Roesseler Liver | Roessler Liver2 | Chen Liver | Wurmbach Liver |
| --- | --- | --- | --- | --- |
| p-value | 3.17E-07 | 5.68E-50 | 7.56E-08 | 7.77E-05 |
| fold change | 2.769 | 1.977 | 1.756 | 1.983 |
| t-Test | 6.692 | 17.942 | 5.5 | 4.231 |
| OverexpressionGene Rank | 496  (in top 4%) | 498  (in top 4%) | 955  (in top 9%) | 1352  (in top 7%) |
