# Supplementary material for: Identification of PAFAH1B3 as Candidate Prognosis Marker and Potential Therapeutic Target for Hepatocellular Carcinoma
Source: Front Oncol. 2021 Aug 19;11:700700. doi: 10.3389/fonc.2021.700700 (PMC8418329; doi:10.3389/fonc.2021.700700)
Supplement: Supplementary Table 2 — The top 50 co-expressed genes of PAFAH1B3 (LinkedOmics). [file Table_2.docx]

**Supplementary Table 2.** The top 50 co-expressed genes of PAFAH1B3 (LinkedOmics).

| Target Gene/Attribute | Spearman Correlation | P-value | FDR |
| --- | --- | --- | --- |
| PAFAH1B3 | 1.00E+00 | 1.00E-68 | 1.00E-64 |
| PRR19 | 7.49E-01 | 5.08E-68 | 5.06E-64 |
| UBE2S | 6.98E-01 | 2.27E-55 | 1.51E-51 |
| SNRPA | 6.85E-01 | 9.19E-53 | 4.58E-49 |
| H2AFX | 6.73E-01 | 2.50E-50 | 8.31E-47 |
| BIRC5 | 6.66E-01 | 7.34E-49 | 1.69E-45 |
| DDX39 | 6.66E-01 | 7.62E-49 | 1.69E-45 |
| PTTG1 | 6.64E-01 | 1.42E-48 | 2.57E-45 |
| MBOAT7 | 6.55E-01 | 8.73E-47 | 1.24E-43 |
| ZNF296 | 6.47E-01 | 1.90E-45 | 2.28E-42 |
| HN1 | 6.47E-01 | 1.95E-45 | 2.28E-42 |
| TACC3 | 6.46E-01 | 3.31E-45 | 3.61E-42 |
| TRIM28 | 6.46E-01 | 3.45E-45 | 3.61E-42 |
| WDR62 | 6.46E-01 | 3.62E-45 | 3.61E-42 |
| PRMT1 | 6.45E-01 | 4.32E-45 | 4.10E-42 |
| KCTD17 | 6.45E-01 | 5.51E-45 | 4.99E-42 |
| TOMM40 | 6.43E-01 | 1.24E-44 | 1.03E-41 |
| CENPM | 6.42E-01 | 1.61E-44 | 1.28E-41 |
| MYBL2 | 6.41E-01 | 2.53E-44 | 1.94E-41 |
| NUDT1 | 6.37E-01 | 1.14E-43 | 7.86E-41 |
| MFSD10 | 6.35E-01 | 3.34E-43 | 2.22E-40 |
| MCRS1 | 6.34E-01 | 4.09E-43 | 2.63E-40 |
| UBE2C | 6.34E-01 | 4.72E-43 | 2.94E-40 |
| C19orf61 | 6.33E-01 | 6.11E-43 | 3.62E-40 |
| ANKRD13D | 6.33E-01 | 6.17E-43 | 3.62E-40 |
| CDC20 | 6.31E-01 | 1.54E-42 | 8.75E-40 |
| AURKB | 6.29E-01 | 2.65E-42 | 1.47E-39 |
| CENPA | 6.24E-01 | 1.76E-41 | 9.22E-39 |
| SNRPD2 | 6.23E-01 | 3.53E-41 | 1.80E-38 |
| TROAP | 6.21E-01 | 6.07E-41 | 3.02E-38 |
| TMEM147 | 6.18E-01 | 1.68E-40 | 7.26E-38 |
| SNRPB | 6.18E-01 | 2.02E-40 | 8.57E-38 |
| TMSB10 | 6.16E-01 | 3.41E-40 | 1.38E-37 |
| CDCA3 | 6.14E-01 | 9.71E-40 | 3.72E-37 |
| LASS5 | 6.13E-01 | 1.06E-39 | 3.98E-37 |
| NRM | 6.13E-01 | 1.15E-39 | 4.18E-37 |
| C9orf140 | 6.13E-01 | 1.12E-39 | 4.14E-37 |
| NR2C2AP | 6.12E-01 | 1.53E-39 | 5.43E-37 |
| RFXANK | 6.12E-01 | 1.63E-39 | 5.69E-37 |
| RNFT2 | 6.12E-01 | 1.76E-39 | 6.04E-37 |
| PIGU | 6.12E-01 | 1.99E-39 | 6.74E-37 |
| GEMIN7 | 6.11E-01 | 2.22E-39 | 7.14E-37 |
| C19orf48 | 6.10E-01 | 3.19E-39 | 9.89E-37 |
| PNKP | 6.09E-01 | 5.06E-39 | 1.53E-36 |
| CCNE1 | 6.09E-01 | 5.62E-39 | 1.67E-36 |
| KIF2C | 6.08E-01 | 7.40E-39 | 2.12E-36 |
| CCNB1 | 6.07E-01 | 1.11E-38 | 3.13E-36 |
| GSK3A | 6.03E-01 | 3.85E-38 | 1.07E-35 |
| PPP1R14B | 6.03E-01 | 4.63E-38 | 1.26E-35 |
| SNRPD1 | 6.01E-01 | 7.30E-38 | 1.94E-35 |
